# 1,8-Dibenzoyl-2,7-dimethoxy­naphthalene

**DOI:** 10.1107/S1600536808007009

**Published:** 2008-04-04

**Authors:** Kosuke Nakaema, Shoji Watanabe, Akiko Okamoto, Keiichi Noguchi, Noriyuki Yonezawa

**Affiliations:** a Department of Organic and Polymer Materials Chemistry, Tokyo University of Agriculture & Technology, Koganei, Tokyo 184-8588, Japan; b Instrumentation Analysis Center, Tokyo University of Agriculture & Technology, Koganei, Tokyo 184-8588, Japan

## Abstract

The mol­ecule of the title compound, C_26_H_20_O_4_, is located on a twofold rotation axis. The two benzoyl groups are situated in an *anti* orientation. The dihedral angle between the mean planes of the phenyl ring and the naphthalene ring system is 80.25 (6)°. The phenyl and carbonyl groups in each benzoyl group are almost coplanar. The mol­ecular packing is stabilized by weak C—H⋯O hydrogen bonds and a π–π stacking inter­action between the phenyl rings [centroid–centroid and inter­planar distances of 3.6383 (10) and 3.294 Å, respectively].

## Related literature

For related literature, see: Cohen *et al.* (2004 ▶); Gore & Henrick (1980 ▶); Nakaema *et al.* (2007 ▶).
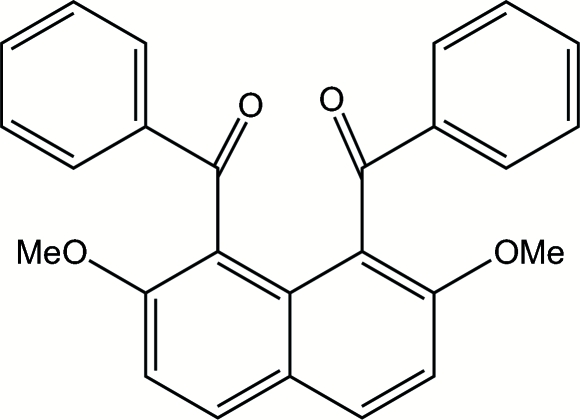


## Experimental

### 

#### Crystal data

C_26_H_20_O_4_
*M* *_r_* = 396.42
Monoclinic, 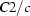

*a* = 13.9677 (4) Å
*b* = 10.2145 (3) Å
*c* = 14.6966 (4) Å
β = 109.711 (2)°
*V* = 1973.95 (10) Å^3^
*Z* = 4
Cu *K*α radiation
μ = 0.72 mm^−1^
*T* = 93 (2) K
0.50 × 0.10 × 0.10 mm

#### Data collection

Rigaku R-AXIS RAPID diffractometer
Absorption correction: numerical (**NUMABS**; Higashi, 1999 ▶) *T*_min_ = 0.838, *T*_max_ = 0.930
17362 measured reflections
1807 independent reflections
1461 reflections with *I* > 2σ(*I*)
*R*_int_ = 0.027

#### Refinement

*R*[*F* ^2^ > 2σ(*F* ^2^)] = 0.039
*wR*(*F* ^2^) = 0.115
*S* = 1.08
1807 reflections
139 parameters
H-atom parameters constrained
Δρ_max_ = 0.19 e Å^−3^
Δρ_min_ = −0.21 e Å^−3^

### 

Data collection: *PROCESS-AUTO* (Rigaku, 1998 ▶); cell refinement: *PROCESS-AUTO*; data reduction: *CrystalStructure* (Rigaku/MSC, 2004 ▶); program(s) used to solve structure: *SIR2004* (Burla *et al.*, 2005 ▶); program(s) used to refine structure: *SHELXL97* (Sheldrick, 2008 ▶); molecular graphics: *ORTEPIII* (Burnett & Johnson, 1996 ▶); software used to prepare material for publication: *SHELXL97*.

## Supplementary Material

Crystal structure: contains datablocks global, I. DOI: 10.1107/S1600536808007009/is2282sup1.cif

Structure factors: contains datablocks I. DOI: 10.1107/S1600536808007009/is2282Isup2.hkl

Additional supplementary materials:  crystallographic information; 3D view; checkCIF report

## Figures and Tables

**Table 1:** Hydrogen-bond geometry (Å, °)

*D*—H⋯*A*	*D*—H	H⋯*A*	*D*⋯*A*	*D*—H⋯*A*
C12—H12⋯O1^i^	0.95	2.60	3.4987 (19)	159
C14—H14*B*⋯O1^ii^	0.98	2.39	3.344 (2)	164

Symmetry codes: (i) 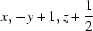; (ii) 
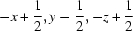
.

**Fig. 1.**
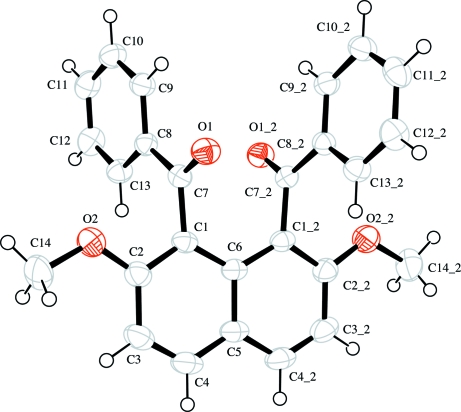
Molecular structure of (I), with the atom-labeling scheme and displacement ellipsoids drawn at the 50% probability level. The symbol "_2" refers to symmetry code: -x, y, -z+1/2.

**Fig. 2.**
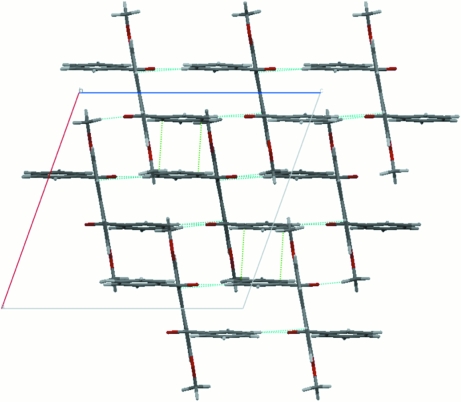
A partial packing diagram of the title compound, viewed down the b axis. The dashed lines indicate hydrogen bonds (blue dashed lines) and π—π stacking interactions (green dashed lines).

## supplementary crystallographic information

### Comment

The molecules with naphthalene frame, especially, *peri*-substituted naphthalenes, have received much attention as unique structured aromatic core compounds for variety of the functional materials. Therefore, structural analyses of *peri-*substituted naphthalenes have been actively performed (Cohen *et al.*, 2004; Gore & Henrick, 1980). Recently, we have reported the structure of 1,8-bis(4-chlorobenzoyl)-2,7-dimethoxynaphthalene (Nakaema *et al.*, 2007). In this paper, the crystallographical structural characteristics of a 1,8-diphenylated naphthalene derivative having two methoxy groups at the 2,7-positions are described as the most simple homolog of the previously reported compound. The title compound was successfully synthesized by regioselective electrophilic aromatic substitution reaction of 2,7-dimethoxynaphthalene with benzoic acid.

*ORTEPIII *(Burnett & Johnson, 1996) plot of title compound is displayed in Fig. 1. The molecule of (I) lies across a crystallographic 2-fold axis so that the asymmetric unit contains one-half of the molecules. Thus, the two benzoyl groups are situated in *anti* orientation. The benzoyl groups are twisted away from the naphthalene moiety, and the dihedral angle is 80.25 (6)°. The torsion angles between the carbonyl groups and the naphthalene ring are -76.73 (18)° [C6—C1—C7—O1], and those between the carbonyl groups and the phenyl groups are 179.75 (15)° [C13—C8—C7—O1].

In the crystal structure, the molecular packing of (I) is mainly stabilized by van der Waals interaction. In addition, the packing of the molecule is stabilized by relatively weak C—H···O hydrogen bonding, namely, C12—H12···O1^i^ [symmetry code: (i) *x*, *-y*+1, *z* + 1/2], C14—H14B···O1^ii^ [symmetry code: (ii) *-x*+1/2, *y* - 1/2, *-z*+1/2], and a π—π stacking interaction. In the packing, the molecules are arranged by C—H···O hydrogen bonding along the *c* axis of the unit cell, and by a π—π stacking interaction perpendicular the *bc* plane of the unit cell (Fig. 2).

### Experimental

The title compound was prepared by electrophilic aromatic diaroylation reaction of 2,7-dimethoxynaphthalene with benzoic acid. White single crystals suitable for X-ray diffraction were obtained by recrystallization from ethanol.

### Refinement

All H atoms were found in a difference map and were subsequently refined as riding atoms, with C—H = 0.95 (aromatic) and 0.98 Å (methyl), and with *U*_iso_(H) = 1.2*U*_eq_(C).

### Crystal data

**Table:**

C_26_H_20_O_4_	*F*_000_ = 832
*M**_r_* = 396.42	*D*_x_ = 1.334 Mg m^−^^3^
Monoclinic, *C*2/*c*	Cu *K*α radiation λ = 1.54187 Å
Hall symbol: -C 2yc	Cell parameters from 10115 reflections
*a* = 13.9677 (4) Å	θ = 3.2–68.1º
*b* = 10.2145 (3) Å	µ = 0.72 mm^−^^1^
*c* = 14.6966 (4) Å	*T* = 93 (2) K
β = 109.711 (2)º	Needle, colorless
*V* = 1973.95 (10) Å^3^	0.50 × 0.10 × 0.10 mm
*Z* = 4	

### Data collection

**Table:**

Rigaku R-AXIS RAPID diffractometer	1807 independent reflections
Radiation source: rotating anode	1461 reflections with *I* > 2σ(*I*)
Monochromator: graphite	*R*_int_ = 0.027
Detector resolution: 10.00 pixels mm^-1^	θ_max_ = 68.2º
*T* = 93(2) K	θ_min_ = 5.5º
ω scans	*h* = −16→16
Absorption correction: numerical(*NUMABS*; Higashi, 1999)	*k* = −12→12
*T*_min_ = 0.838, *T*_max_ = 0.930	*l* = −17→17
17362 measured reflections	

### Refinement

**Table:**

Refinement on *F*^2^	Hydrogen site location: difference Fourier map
Least-squares matrix: full	H-atom parameters constrained
*R*[*F*^2^ > 2σ(*F*^2^)] = 0.039	*w* = 1/[σ^2^(*F*_o_^2^) + (0.0579*P*)^2^ + 0.9602*P*] where *P* = (*F*_o_^2^ + 2*F*_c_^2^)/3
*wR*(*F*^2^) = 0.115	(Δ/σ)_max_ < 0.001
*S* = 1.08	Δρ_max_ = 0.19 e Å^−^^3^
1807 reflections	Δρ_min_ = −0.21 e Å^−^^3^
139 parameters	Extinction correction: SHELXL97 (Sheldrick, 2008), Fc^*^=kFc[1+0.001xFc^2^λ^3^/sin(2θ)]^-1/4^
Primary atom site location: structure-invariant direct methods	Extinction coefficient: 0.00121 (18)
Secondary atom site location: difference Fourier map	

### Special details

**Table:**

Geometry. All e.s.d.'s (except the e.s.d. in the dihedral angle between two l.s. planes) are estimated using the full covariance matrix. The cell e.s.d.'s are taken into account individually in the estimation of e.s.d.'s in distances, angles and torsion angles; correlations between e.s.d.'s in cell parameters are only used when they are defined by crystal symmetry. An approximate (isotropic) treatment of cell e.s.d.'s is used for estimating e.s.d.'s involving l.s. planes.
Refinement. Refinement of *F*^2^ against ALL reflections. The weighted *R*-factor *wR* and goodness of fit *S* are based on *F*^2^, conventional *R*-factors *R* are based on *F*, with *F* set to zero for negative *F*^2^. The threshold expression of *F*^2^ > σ(*F*^2^) is used only for calculating *R*-factors(gt) *etc*. and is not relevant to the choice of reflections for refinement. *R*-factors based on *F*^2^ are statistically about twice as large as those based on *F*, and *R*- factors based on ALL data will be even larger.

### Fractional atomic coordinates and isotropic or equivalent isotropic displacement parameters (Å^2^)

**Table:**

	*x*	*y*	*z*	*U*_iso_*/*U*_eq_	
O1	0.11104 (8)	0.59739 (10)	0.22559 (7)	0.0399 (3)	
O2	0.26822 (8)	0.39816 (11)	0.37874 (9)	0.0530 (4)	
C1	0.09424 (11)	0.39621 (13)	0.29711 (10)	0.0325 (3)	
C2	0.18262 (12)	0.32529 (15)	0.33814 (11)	0.0393 (4)	
C3	0.18231 (14)	0.18652 (16)	0.33535 (12)	0.0470 (4)	
H3	0.2436	0.1385	0.3626	0.056*	
C4	0.09246 (14)	0.12359 (15)	0.29280 (11)	0.0463 (4)	
H4	0.0919	0.0306	0.2919	0.056*	
C5	0.0000	0.19109 (19)	0.2500	0.0383 (5)	
C6	0.0000	0.33146 (18)	0.2500	0.0319 (4)	
C7	0.10671 (10)	0.54368 (14)	0.29822 (10)	0.0313 (3)	
C8	0.11341 (10)	0.61894 (13)	0.38633 (10)	0.0316 (3)	
C9	0.12438 (11)	0.75478 (14)	0.38552 (11)	0.0368 (4)	
H9	0.1276	0.7970	0.3291	0.044*	
C10	0.13055 (12)	0.82794 (16)	0.46611 (12)	0.0431 (4)	
H10	0.1384	0.9203	0.4652	0.052*	
C11	0.12535 (12)	0.76686 (17)	0.54845 (12)	0.0448 (4)	
H11	0.1296	0.8173	0.6040	0.054*	
C12	0.11397 (12)	0.63233 (17)	0.54997 (11)	0.0447 (4)	
H12	0.1102	0.5906	0.6064	0.054*	
C13	0.10816 (11)	0.55857 (15)	0.46934 (10)	0.0376 (4)	
H13	0.1006	0.4662	0.4707	0.045*	
C14	0.36309 (13)	0.3343 (2)	0.42144 (14)	0.0592 (5)	
H14A	0.4171	0.3999	0.4447	0.071*	
H14B	0.3771	0.2783	0.3733	0.071*	
H14C	0.3606	0.2805	0.4758	0.071*	

### Atomic displacement parameters (Å^2^)

**Table:**

	*U*^11^	*U*^22^	*U*^33^	*U*^12^	*U*^13^	*U*^23^
O1	0.0505 (6)	0.0371 (6)	0.0351 (6)	−0.0020 (4)	0.0185 (5)	0.0020 (4)
O2	0.0393 (6)	0.0456 (7)	0.0658 (8)	0.0087 (5)	0.0068 (5)	−0.0046 (6)
C1	0.0421 (8)	0.0273 (7)	0.0311 (7)	0.0034 (6)	0.0161 (6)	0.0006 (5)
C2	0.0446 (9)	0.0366 (8)	0.0366 (8)	0.0058 (6)	0.0134 (7)	−0.0009 (6)
C3	0.0591 (10)	0.0375 (9)	0.0442 (9)	0.0160 (7)	0.0170 (8)	0.0021 (7)
C4	0.0708 (12)	0.0281 (8)	0.0428 (9)	0.0076 (7)	0.0227 (8)	0.0014 (6)
C5	0.0583 (13)	0.0268 (10)	0.0342 (11)	0.000	0.0212 (10)	0.000
C6	0.0454 (11)	0.0266 (9)	0.0278 (10)	0.000	0.0177 (9)	0.000
C7	0.0307 (7)	0.0306 (7)	0.0330 (8)	0.0011 (5)	0.0116 (6)	0.0022 (6)
C8	0.0300 (7)	0.0312 (7)	0.0333 (8)	−0.0003 (5)	0.0101 (6)	−0.0011 (6)
C9	0.0415 (8)	0.0320 (7)	0.0367 (8)	0.0012 (6)	0.0130 (6)	0.0018 (6)
C10	0.0453 (9)	0.0353 (8)	0.0478 (10)	0.0000 (6)	0.0143 (7)	−0.0069 (7)
C11	0.0426 (9)	0.0515 (10)	0.0415 (9)	−0.0001 (7)	0.0157 (7)	−0.0136 (7)
C12	0.0493 (9)	0.0534 (10)	0.0356 (9)	−0.0040 (7)	0.0199 (7)	−0.0013 (7)
C13	0.0408 (8)	0.0359 (8)	0.0381 (8)	−0.0025 (6)	0.0159 (6)	0.0013 (6)
C14	0.0465 (10)	0.0653 (11)	0.0578 (11)	0.0221 (9)	0.0070 (8)	−0.0151 (9)

### Geometric parameters (Å, °)

**Table:**

O1—C7	1.2197 (16)	C8—C9	1.396 (2)
O2—C2	1.3633 (19)	C9—C10	1.378 (2)
O2—C14	1.4194 (19)	C9—H9	0.9500
C1—C2	1.382 (2)	C10—C11	1.385 (2)
C1—C6	1.4264 (17)	C10—H10	0.9500
C1—C7	1.5158 (19)	C11—C12	1.384 (2)
C2—C3	1.418 (2)	C11—H11	0.9500
C3—C4	1.360 (2)	C12—C13	1.383 (2)
C3—H3	0.9500	C12—H12	0.9500
C4—C5	1.4110 (19)	C13—H13	0.9500
C4—H4	0.9500	C14—H14A	0.9800
C5—C6	1.434 (3)	C14—H14B	0.9800
C7—C8	1.4814 (19)	C14—H14C	0.9800
C8—C13	1.3908 (19)		
			
C2—O2—C14	119.52 (13)	C13—C8—C7	122.02 (13)
C2—C1—C6	120.72 (14)	C9—C8—C7	118.88 (13)
C2—C1—C7	115.70 (13)	C10—C9—C8	120.44 (14)
C6—C1—C7	123.36 (13)	C10—C9—H9	119.8
O2—C2—C1	115.29 (13)	C8—C9—H9	119.8
O2—C2—C3	123.51 (14)	C9—C10—C11	120.00 (15)
C1—C2—C3	121.19 (15)	C9—C10—H10	120.0
C4—C3—C2	118.62 (15)	C11—C10—H10	120.0
C4—C3—H3	120.7	C12—C11—C10	120.09 (15)
C2—C3—H3	120.7	C12—C11—H11	120.0
C3—C4—C5	122.54 (15)	C10—C11—H11	120.0
C3—C4—H4	118.7	C13—C12—C11	120.05 (15)
C5—C4—H4	118.7	C13—C12—H12	120.0
C4^i^—C5—C4	121.50 (19)	C11—C12—H12	120.0
C4^i^—C5—C6	119.25 (10)	C12—C13—C8	120.31 (14)
C4—C5—C6	119.25 (10)	C12—C13—H13	119.8
C1—C6—C1^i^	124.75 (17)	C8—C13—H13	119.8
C1—C6—C5	117.62 (9)	O2—C14—H14A	109.5
C1^i^—C6—C5	117.62 (9)	O2—C14—H14B	109.5
O1—C7—C8	121.63 (13)	H14A—C14—H14B	109.5
O1—C7—C1	118.49 (12)	O2—C14—H14C	109.5
C8—C7—C1	119.88 (12)	H14A—C14—H14C	109.5
C13—C8—C9	119.10 (13)	H14B—C14—H14C	109.5
			
C14—O2—C2—C1	−179.05 (14)	C4—C5—C6—C1^i^	177.70 (9)
C14—O2—C2—C3	−0.3 (2)	C2—C1—C7—O1	97.99 (16)
C6—C1—C2—O2	178.29 (11)	C6—C1—C7—O1	−76.73 (16)
C7—C1—C2—O2	3.43 (18)	C2—C1—C7—C8	−81.97 (16)
C6—C1—C2—C3	−0.5 (2)	C6—C1—C7—C8	103.32 (14)
C7—C1—C2—C3	−175.33 (14)	O1—C7—C8—C13	179.76 (13)
O2—C2—C3—C4	−179.86 (14)	C1—C7—C8—C13	−0.29 (19)
C1—C2—C3—C4	−1.2 (2)	O1—C7—C8—C9	0.4 (2)
C2—C3—C4—C5	1.1 (2)	C1—C7—C8—C9	−179.69 (12)
C3—C4—C5—C4^i^	−179.31 (17)	C13—C8—C9—C10	0.4 (2)
C3—C4—C5—C6	0.69 (17)	C7—C8—C9—C10	179.81 (13)
C2—C1—C6—C1^i^	−177.80 (14)	C8—C9—C10—C11	−0.3 (2)
C7—C1—C6—C1^i^	−3.35 (9)	C9—C10—C11—C12	0.0 (2)
C2—C1—C6—C5	2.20 (14)	C10—C11—C12—C13	0.2 (2)
C7—C1—C6—C5	176.65 (9)	C11—C12—C13—C8	−0.2 (2)
C4^i^—C5—C6—C1	177.70 (9)	C9—C8—C13—C12	−0.1 (2)
C4—C5—C6—C1	−2.30 (9)	C7—C8—C13—C12	−179.53 (13)
C4^i^—C5—C6—C1^i^	−2.30 (9)		

Symmetry codes: (i) −*x*, *y*, −*z*+1/2.

### Hydrogen-bond geometry (Å, °)

**Table:**

*D*—H···*A*	*D*—H	H···*A*	*D*···*A*	*D*—H···*A*
C12—H12···O1^ii^	0.95	2.60	3.4987 (19)	159
C14—H14B···O1^iii^	0.98	2.39	3.344 (2)	164

Symmetry codes: (ii) *x*, −*y*+1, *z*+1/2; (iii) −*x*+1/2, *y*−1/2, −*z*+1/2.
